# Nanoparticles for autoimmune diseases: advancing diagnostics and therapeutic solutions

**DOI:** 10.1038/s41401-026-01794-w

**Published:** 2026-05-04

**Authors:** Elaine Xue Ning Ong, Chester Yan Jie Ng, Lu-lei Cao, Xuexin Li, Pan P. Li, Nguan Soon Tan, Zehuan Liao, Yan Zhao

**Affiliations:** 1 School of Biological Sciences, Nanyang Technological University, Singapore, 637551 Singapore; 2 Graduate School, Beijing University of Chinese Medicine, Beijing, 100029 China; 3 Department of Physiology and Pharmacology, Karolinska Institutet, Stockholm, 17165 Sweden; 4 Department of Psychiatry and Behavioral Sciences, Division of Neurobiology, Johns Hopkins University School of Medicine, Baltimore, MD 21205 USA; 5 Lee Kong Chian School of Medicine, Nanyang Technological University, Singapore, 308232 Singapore; 6 Department of Microbiology, Tumor and Cell Biology, Karolinska Institutet, Stockholm, 17177 Sweden

**Keywords:** autoimmune diseases, nanoparticles, diagnostic tools, therapeutic tools

## Abstract

Autoimmune diseases pose a significant challenge to modern medicine due to their complicated and poorly understood mechanisms, which hinder effective diagnosis and treatment. The rising global incidence of autoimmune disorders is projected to place increasing strain on healthcare systems and financial resources. In response, nanotechnology has emerged as a promising avenue for both the diagnosis and treatment of these conditions. Among various nano-technological approaches, nanoparticles have garnered particular attention due to their advantageous properties including biocompatibility, enhanced drug bioavailability and efficient permeability across biological membranes. Furthermore, their unique characteristics such as magnetic responsiveness, anti-inflammatory, and antimicrobial capabilities, enable precise drug delivery and improved therapeutic outcomes, as well as the potential for earlier disease detection. Despite these promising developments, the clinical translation of nanoparticle-based strategies faces challenges including concerns regarding their stability in vivo and the need for further research to validate their safety and efficacy. While current diagnostic tools remain limited, certain nanoparticles have already received approval from the US Food and Drug Administration, demonstrating their potential for clinical application. This review aims to highlight the recent advances in use of nanoparticles for the diagnosis and treatment of autoimmune diseases, and to explore their prospective role in future clinical practice.

## Introduction

Globally, the number of patients diagnosed with autoimmune diseases is rising, driven by factors such as environmental exposures, genetic predisposition, and stress [1]. Autoimmune diseases are chronic inflammatory conditions, and the National Institute of Environmental Health Sciences recognizes more than 80 distinct autoimmune disorders, including Graves’ disease, multiple sclerosis (MS), systemic lupus erythematosus (SLE), type 1 diabetes mellitus (T1D), and rheumatoid arthritis (RA) [2]. Studies estimate that nearly 10% of the global population is affected by one or more autoimmune diseases, resulting in substantial impairment in quality of life, increased morbidity, and higher mortality [3]. These numbers continue to increase, with a reported estimated annual rise of 19.1% worldwide [1]. In parallel, patients face a significant financial burden and medical complications associated with conventional therapies [3]. Together, the growing prevalence and long-term complications of autoimmune diseases underscore the need for improved strategies that reduce chronic disease burden for both patients and society.

A major challenge in managing autoimmune diseases is early diagnosis. Because early manifestations often resemble non-specific inflammatory symptoms, timely and accurate diagnosis remains difficult [4]. In many cases, autoimmune diseases are only recognized after substantial, and sometimes irreversible, tissue injury has occurred, which contributes to chronic complications and worse outcomes [4]. Thus, improved tools for earlier detection are needed. Beyond diagnosis, another major challenge is improving treatment and preventing disease progression in individuals with established autoimmune diseases. Currently, therapies rely largely on pharmacological agents that curb inflammation by targeting immunoregulatory pathways, including Janus Kinase (JAK) inhibitors and other intracellular tyrosine kinases [5, 6]. However, systemic administration can limit delivery to target tissues due to pharmacodynamics and pharmacokinetics constraints, reducing effective bioavailability at sites of inflammation [7]. While intravenous delivery can increase systemic exposure, it may also introduce complications such as abscess formation, neurovascular injury, muscle fibrosis, and persistent pain at the injection site [7, 8].

Nanoparticles (NP) offer a potential alternative approach because they can be engineered for targeted delivery, controlled release, and tissue-specific accumulation. In addition to improving drug delivery, NPs can be designed to promote immune self-tolerance and confer added functionality depending on surface coatings and material properties [9, 10]. Their small size may also enable them to traverse selective biological barriers and support lower effective dosing, thereby potentially reducing off-target exposure and adverse effects [11]. Given these advantages, NP-based strategies warrant further exploration for autoimmune disease diagnosis and therapy.

Hence, in this review, we consolidate recent advances in commonly used NP platforms with demonstrated diagnostic and therapeutic potential in autoimmune disease, and outline perspectives and priorities for future research.

## Characteristics of nanoparticles

Currently, several biomarkers, including antinuclear antibody (ANA), antiphospholipid antibodies and auto-antibodies (e.g., anti-Ro/SSA and anti-La/SSB), are used to support the diagnosis of autoimmune diseases [12]. These biomarkers are assessed using serology, urine, cerebrospinal fluids, body fluids and other inflammatory indicators [13]. However, there is no single test that reliably diagnoses all autoimmune diseases because different conditions involve distinct biomarker profiles [14]. Moreover, many existing diagnostic approaches have limited sensitivity, reducing their ability to detect early molecular changes before tissue damage occurs [15]. One potential solution is the use of NP-based biosensors, which enable impedimetric, colorimetric, and electrochemical detection, and may improve sensitivity for early molecular changes [15]. NPs with redox properties such as gold- and iron-based NPs, are often incorporated as imaging contrast agents or signal-generating components conjugated to detectors, producing measurable output upon binding to disease-associated antibodies [15]. In parallel, advances in NP engineering have enabled therapeutic or diagnostic cargo to be delivered across physiological barriers with high specificity, potentially improving pharmacokinetics, bioavailability, and plasma half-life by shielding payloads from enzymatic degradation and enabling sustained release [16].

Nanoparticles are typically defined as engineered particles ranging from 1–100 nanometers (nm) in size [17, 18]. They can be designed to deliver drugs to target specific tissues or diseased cells, while minimizing interactions with healthy cells, thereby reducing off-target effects [19]. Compared with conventional formulations, NPs can provide more controlled drug release, and higher delivery precision, which may increase bioavailability and prolonged therapeutic exposure [19, 20]. Because NPs differ widely in size, shape, material composition, and optical/electromagnetic properties [19], selecting an appropriate platform requires consideration of factors such as particle size for target access, carrier biocompatibility to reduce toxicity, and stability to prevent premature aggregation or degradation [19]. Importantly, interactions between NPs and target tissues depend strongly on surface chemistry. Surface modifications can alter cellular uptake, biodistribution, and payload release efficiency.

**Fig. 1:**
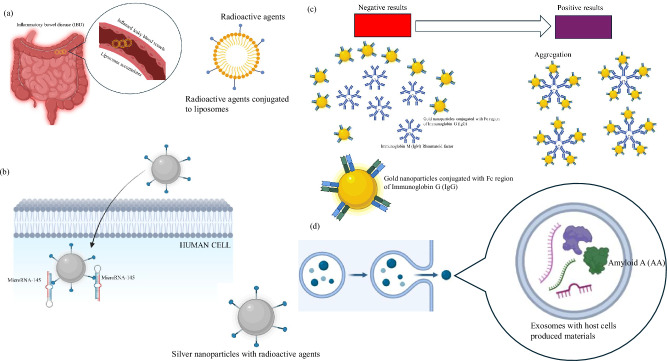
An overview of various types of nanoparticles used in medical diagnosis*.* **a** Liposomes conjugated with radioactive agents aggregate at inflamed sites and emit signals to indicate inflammatory bowel disease (IBD). **b** Silver nanoparticles conjugate with radioactive agents can transverse selective membrane and recognize microRNA-145 in MS patients, improving probe sensitivity. **c** Gold nanoparticles conjugated with the Fc region of immunoglobin G (IgG) recognize pathological immunoglobin M (IgM), a Rheumatoid Factor (RF), producing a visible color change from red to purple, suggestive of rheumatoid arthritis (RA). **d** Exosomes released by pathological cells encapsulate pathological biomarkers such as amyloid A (AA).

Incorporating NPs into autoimmune disease management may improve both diagnosis and therapy (Fig. 1). Timing is critical [21, 22], and delayed diagnosis increases the likelihood that irreversible complications have already developed [23, 24]. Patients frequently seek medical attention only after symptoms substantially impair quality of life, by which point tissue injury may be established, such as lupus nephritis in SLE, pericarditis in RA, or thromboembolic complications in Crohn’s disease [25, 26]. NP-enabled approaches could therefore support earlier detection, even in minimally symptomatic or asymptomatic stages, and improve treatment outcomes.

Nanoparticles can be categorized as organic, inorganic, or carbon-based, based on their materials and physicochemical properties. Modulating parameters such as size, shape, surface charge, stiffness, and surface chemical composition can alter interactions with immune cells, including T cells, dendritic cells (DCs), and macrophages, expanding their applicability for immunological regulation [27].

As immunomodulatory agents, NPs can serve as adjuvants that support drug delivery or selective influence immune cell subpopulations [28]. Specificity for target tissues can be increased through surface modification such as conjugation of ligands, antibodies or peptides, which can enhance efficacy while reducing toxicity [29]. Such designs may also promote anti-inflammatory programs, for example, by supporting M2 macrophage polarization, thereby helping to re-establish immune tolerance while preserving the ability to respond to non-self signals [28].

### Size of nanoparticles

Nanoparticles are attractive because their small size can enable passage across highly selective barriers such as the blood brain barrier (BBB). However, reducing particle size increases surface area-to-volume ratio, which can strengthen interparticle interactions and promote agglomeration/aggregation [17]. Aggregation can change surface electron-transfer properties and expose catalytic interfaces that accelerate redox cycling [30, 31], potentially increasing oxidative stress and nanotoxicity even when the core material and chemical composition are unchanged [32, 33]. In turn, aggregated NPs may increase reactive oxygen species (ROS) generation, alter biodistribution and accumulation sites, and disrupt redox homeostasis in immune cells and tissues. Excess ROS can damage biomacromolecules, promote pro-inflammatory cytokine release, and skew immune differentiation, potentially exacerbating autoimmune inflammation and tissue injury [34, 35]. In vivo, NP size also influences biodistribution, interstitial diffusion, and uptake mechanisms such as endocytosis versus phagocytosis, and systemic clearance [27]. Although smaller NPs often elicit stronger stimulation of antigen-presenting cells (APCs) in vivo, chemical composition and surface modifications can substantially modulate these effects [27].

### Surface charge of nanoparticles

Surface charge influences NP interactions with biological membranes and immune molecules, and can affect clearance rates. Cationic, anionic, neutral, and slightly anionic NPs have been reported to be cleared from fastest to slowest, respectively [36]. Cationic NPs are often internalized more readily through electrostatic interactions with negatively charged cell membranes, but they may also trigger undesired inflammatory responses [37]. In contrast, anionic NPs, despite typically slower uptake, have been associated with Type 1 helper (Th1)-type immune responses that can support anti-inflammatory mechanisms in specific contexts [37]. Notably, faster internalization does not necessarily translate to greater functional potency. One study found that although cationic NPs were taken up more rapidly, vesicular trans-monolayer trafficking was impeded, whereas anionic NP exhibited slower uptake but more efficient cortical transport [37].

## Nanoparticles for diagnosis of autoimmune diseases

### Metal-based nanoparticles

Medical imaging, primarily X-ray, CT-scans, and Magnetic Resonance Imaging (MRI), is widely used to support the diagnosis of autoimmune disease and to differentiate disease stages. These modalities are non-invasive and can penetrate soft tissues to reveal structural and molecular abnormalities [38]. Among them, MRI provides detailed information on soft tissues and vascular features, however, its relatively low sensitivity often necessitates the use of metal-based NPs as contrast agents to improve visualization and diagnostic performance [39].

Metal-based NPs such as silver, cobalt, titanium dioxide, aluminum oxide, zinc oxide, iron oxide and gold NPs, can serve diagnostic and therapeutic roles due to their distinct physicochemical properties. Beyond acting as carriers, certain metal-based NPs exhibit intrinsic anti-inflammatory activity and can function as therapeutic agents without encapsulating additional drugs or requiring external stimuli such as heat, magnetic fields, or light [40]. In addition, magnetic, photothermal, and plasmonic properties can be exploited for non-invasive imaging, which may enable earlier detection of autoimmune-related pathology [41].

Nevertheless, not all metal-based NPs are suitable for autoimmune applications. Potential toxicity, including ROS production leading to oxidative stress and inflammation, can damage cellular components such as DNA, protein and membranes [42].

#### Iron oxide nanoparticles (IONPs)

Gadolinium-based contrast agents (GBCA) remain widely used in MRI. However, concerns persist regarding potential toxicity, including unpredictable release of free Gd^3+^ ions [43], and long-term retention in the body, which may contribute to adverse outcomes such as nephrogenic systemic fibrosis [39], renal complications [44] and possible brain deposition [45].

Iron oxide nanoparticles (IONPs), among the most extensively studied magnetic nanoparticles (MNPs), are promising alternatives to GBCA. IONPs are generally considered biodegradable and can be cleared from the body [45]. They can also be directed using external magnetic fields and engineered to detect biomarkers by producing electromagnetic, photonic, or acoustic signals upon sensing specific pathological targets, enabling potentially sensitive and affordable detection [38].

The hydrodynamic diameter of IONPs can be tuned to generate Standard IONPs (0–180 nm), Superparamagnetic Iron Oxide NP (SPIONPs, 10–50 nm), Very Small Superparamagnetic Iron Oxide NP (VSPIONPs, <10 nm) [46] and Ultrasmall Iron Oxide NP (USIONPs, <5 nm) [47]. In one study, classic monocytes internalized superparamagnetic fluorescent IONPs more efficiently than non-classical monocytes, supporting the concept that differential NP uptake by immune subsets can be leveraged for disease characterization. This is highly beneficial as it offers ease of diagnosis through the observation of monocyte population and its accumulation of NP via fluorescence microscopy [46]. Importantly, polyacrylate-coated IONPs did not appear to impair monocyte migration toward inflammatory sites, enabling in vivo accumulation for monitoring of early tissue injury [46]. Notably, MRI identified 188 USIONP-positive lesions of which 144 were gadolinium negative [46], suggesting that USIONPs can reveal lesions missed by GBCA [46]. Combining USIONPs with gadolinium may therefore improve lesion detection in MS, although improved detection does not necessarily equate to improved diagnostic accuracy, thus necessitating complementary approaches.

Further work demonstrates that modifying IONP core size and coating including thinner coating or alternative coating materials can enhance T1-weighted MRI signals and positive contrast in vivo [44]. Examples include non-toxic Manganese (Mn)-doped IONPs yielding signal enhancement in tumor-bearing mice, Copper(Cu)-doped IONPs (Cu4-NPs) conjugated with RGD increasing cardiac signal, and poly(acrylic acid)-poly(methacrylic acid) IONPs (PMAA-PTTM-IONPs) strengthening renal and hepatic signals [44]. Although these studies were not conducted in autoimmune models, they support the feasibility of using optimized IONP formulations to amplify T1 signals and capture subtle pathological changes indicative of early autoimmune disease, including organ-specific conditions such as T1D [4, 6].

For example, early T1D detection has been explored using ferumoxtran (a USIONP) to image leukocyte infiltration in the pancreas, leveraging inflammation-associated vascular leakiness, a fundamental hallmark of autoimmune illness caused by continuous inflammation [38]. Leukocytes engulf USIONPs and accumulate at inflamed pancreatic sites, producing detectable signals that could enable identification of asymptomatic individuals before overt diabetes [38].

Beyond early autoimmune detection, NP-based imaging may help identify complications that are often under-recognized. Cardiovascular disease (CVD) is a major comorbidity in autoimmune diseases and is commonly assessed using a non-invasive diagnostic technique, cardiovascular magnetic resonance imaging (CMR), typically with gadolinium enhancement [48]. Given gadolinium-associated risks, biodegradable IONPs may represent a safer alternative in some contexts [49]. USIONPs can infiltrate inflamed tissue via macrophage uptake and then accumulate in the myocardium, enabling detection of active myocardial inflammation [47, 50]. Using USIONP-enhanced CMR, persistent inflammation in chronic infarcted and remote myocardium after myocardial infarction has been reported, supporting feasibility for inflammation imaging [47]. USIONPs may also be advantageous in patients with chronic kidney disease who are at risk of nephrogenic systemic fibrosis [47] due to its small size, reducing risks of damaging the blood vessels in kidneys and other health implications. This has potential relevance for SLE, where CVD risk can be high even in the absence of overt symptoms, and earlier detection could improve prognosis [48].

Standard MRI may be insufficient for risk assessment or very early diagnosis in central nervous system (CNS) autoimmune diseases, particularly before substantial disability develops [51, 52]. VSPIONPs may address this limitation due to their smaller size relative to USIONPs and SPIONs, enabling tracking of phagocytic cells in vivo, detection of blood-brain barrier (BBB) disruption in experimental autoimmune encephalomyelitis (EAE) models, and visualization of early changes at the blood-cerebrospinal fluid barrier (BCSFB) that may not be evident with gadolinium agents [51]. Citrate-stabilized VSPIONPs with small hydrodynamic diameter (e.g., ~7 nm) exhibit high permeability and can help identify early hallmarks of CNS inflammation. However, histological methods for identifying and differentiating VSPIONPs remain limited [51]. To address this, europium (Eu)-doped VSPIONPs have been explored because Eu fluorescence can aid detection. In addition, antibody conjugation can improve biomarker specificity and potentially reduce false positives [51].

Collectively, IONPs show potential as clinically useful contrast agents due to their biodegradability, suitability for patients at risk from gadolinium exposure, and ability to reveal active inflammation that may otherwise be missed, thereby informing treatment planning and potentially slowing disease progression.

#### Gold nanoparticles (GNPs)

**Fig. 2:**
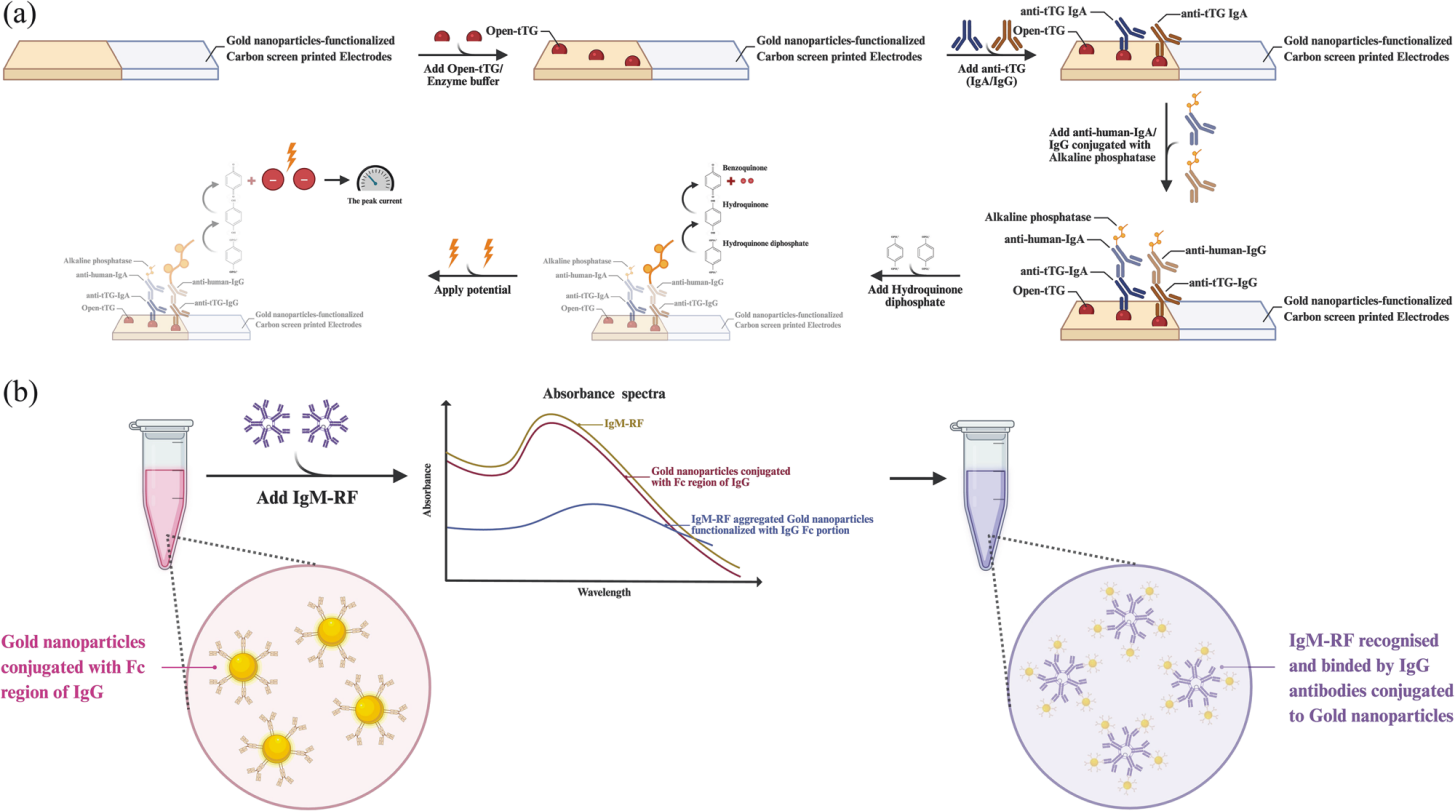
An overview of the medical applications of GNPs*.* **a** GNPs-functionalized carbon screen-printed electrodes with immobilized transglutaminase bind to anti-tissue transglutaminase antibodies (anti-tTG), producing amperometric signals illustrating the “specific binding of serum antibodies to immobilized antigen” concept for signal amplification. **b** GNP-based biosensor enables one-step colorimetric detection for RA by using plasma biomarkers.

Gold NPs (GNPs) are attractive for autoimmune disease diagnosis and potentially in treatment due to their inertness, high biocompatibility, low cytotoxicity, and stability. Importantly, GNPs are also reported to exhibit anti-inflammatory and antioxidant properties (Fig. 2) [15]. GNPs have been employed as transducers in biosensors, giving a very successful one-step colorimetric detection for RA by assessing the presence of RA biomarkers in patients' blood plasma, even at low concentration [53]. After testing, results produced are highly reliable and identified as a color change from red to purple [53]. This color change is induced through formation of a complex comprising modified highly sensitive GNPs conjugated with IgG Fc region to pathological RA immunoglobulin M (IgM) [53]. Upon binding, this stimulates localized surface plasmon resonance (LSPR), a unique optical characteristic of GNPs, which induces a change in wavelength absorption and hence, visible optical changes [53]. This complex can be confirmed by viewing it under dynamic light scattering (DLS) and atomic force microscopy (AFM) [53]. Overall, such rapid and sensitive platforms may support earlier diagnosis compared with conventional methods with lower sensitivity [54].

A limitation in RA diagnosis is distinguishing early-stage joint changes from normal variation using standard imaging (e.g., X-ray and CT), because early abnormalities can be subtle [55]. This may be addressed by using highly sensitive GNP-enabled biosensors that quantify immune cell abundance, such as monocytes or macrophages, and abnormal pro-inflammatory cytokines, IL-6, TNF-α, and IL-17 [55], rather than relying solely on structural imaging changes. Furthermore, GNPs can be involved in the diagnosis of celiac disease whereby an electrochemical platform encompassing “GNPs-functionalized carbon screen-printed electrodes on transglutaminase immobilized” can bind to anti-tissue transglutaminase antibodies (anti-tTG), producing amperometric signals which illustrates the “specific binding of serum antibodies to immobilized antigen” to strengthen signals and hence, a potential detection methodology for MS diagnosis [15]. Together, these findings highlight the potential for GNPs to complement existing diagnostics and enable simple, user-friendly testing platforms that support earlier detection.

#### Silver nanoparticle (AgNPs)

Silver NPs (AgNPs) are a cost-effective option for probe modification to improve electroanalytical sensitivity and produce more significant results [56]. AgNPs are also well known for their anti-microbial properties [56], which could reduce the risk of infection while increasing the sensitivity of probes to detect specific biomarkers [57]. For example, AgNP-based signal amplification via fluorescence enhancement has been used to detect microRNA-145 biomarker in MS patients significantly improving the sensitivity of electrochemiluminescence (ECL) assays [58]. Beyond metal-based systems, lipid-based NPs offer strong biocompatibility and tunable pharmacokinetics, making them attractive candidates for clinical translation.

### Lipid-based nanoparticles

#### Liposomes

Liposomes are highly biocompatible NPs composed of an outer phospholipid bilayer resembling human biological membranes, with a hydrophobic core to facilitate the delivery of both soluble and insoluble components to target site [59]. A recent study utilizing DiR-labeled liposomes for active targeting to visualize regions of severe inflammation has been successful in the detection of inflammatory sites in patients with Inflammatory Bowel Disease (IBD) [60]. This approach leverages the tendency of liposomes to accumulate at inflamed sites and emit signals that may reflect disease progression [60]. Technetium-99m (⁹⁹ᵐTc)-labeled liposomes used in the IBD diagnostic experiments markedly prolonged the retention of radioactive signals in the intestine for over 24 h, allowing clear visualization of inflamed intestinal regions in colitis rats. They demonstrated high target specificity by predominantly accumulating at the inflammation sites while minimizing uptake in non-target organs such as the spleen and liver, thereby reducing renal accumulation [60]. This suggests efficient clearance from the body, decreasing chances of plausible health complications caused by accumulation of liposomes within the bodies while producing strengthened signals for better diagnostic results [60].

Similarly, liposomes conjugated with ART-1 peptide and encapsulating Cy7 dye were efficiently captured within arthritic rat synovial spaces and generated sustained fluorescence signals [61]. Signals in joints and major organs (brain, spleen, liver) became undetectable after 48 h, suggesting efficient clearance and a favorable safety profile [61]. Overall, liposomes may therefore be useful for autoimmune disease diagnosis.

#### Exosomes

Exosomes are also promising candidates for early detection of autoimmune disease [62]. They encapsulate various biological molecules such as lipids, proteins, messenger RNA (mRNA), and microRNA (miRNA) to facilitate intercellular and interorgan communication, discharge of cellular waste, and cellular homeostasis [63]. Exosome cargo includes approximately 4563 proteins, 1639 mRNAs, and 764 miRNAs, can effectively transport essential molecular signals through autocrine, paracrine, and endocrine pathways, thereby facilitating intercellular communication [63]. Studies have reported significant differences in concentration of components found within the compartment of exosomes that differentiates RA patients from normal healthy people which aid early detection of RA based on analyzing the varying concentration of plausible pathological contents within exosomes such as Hotair, MEG9, miRNA-17 and more [62]. In addition to identifying rheumatoid factor (RF) and/or anti-cyclic citrullinated peptide (anti-CCP) autoantibodies for the diagnosis of RA, recent studies have revealed that circulating exosomal miRNAs, particularly exomiR-151a-3p, exomiR-199a-5p, exomiR-370-3p, and exomiR-769-5p, are closely associated with RA pathogenesis, offering promising diagnostic biomarkers for early detection of RA [64]. Moreover, elevated levels of miR-132 observed in RA patients compared to healthy individuals further underscore the potential of integrating multiple biomarkers for improved diagnostic accuracy. Additionally, reduced levels of miR-6089, a miRNA that directly targets toll-like receptor 4 (TLR4) and modulates LPS/TLR4-mediated inflammatory responses, highlight its potential as another plausible biomarker for RA [64]. By analyzing the contents within exosomes, this can help to increase specificity in identifying and differentiating between autoimmune diseases.

Beyond diagnosis, exosomes also hold potential for monitoring drug efficacy, as they are secreted by virtually all cell types and can be collected to assess a patient’s physiological condition. Unlike conventional diagnostic NPs, exosomes are naturally present in nearly all body fluids, enabling non-invasive sample collection and minimizing health risks such as infection or inflammation at injection sites [62]. Low sample exosomes can be concentrated and purified via nanoporous membrane-based resonators or iTEARs for a high yield of exosomes [62]. Nanoporous membrane-based resonators or iTEARs are highly sensitive for the detection of various pathological biomarkers on exosomes, making them a favorable diagnostic tool for consistent disease monitoring and evaluating the efficacy of therapeutic drugs given to patients with SS [62]. The external biomarkers present on the cell surface and protein components found within the lumen of exosomes are significant diagnostic biomarkers as they are highly reflective of the biological state of parent cells [65]. An experiment utilizing exosome-derived biomarkers amyloid A (AA), lymphatic vessel endothelial hyaluronic acid receptor-1 (LYVE-1), and long non-coding RNA HOX transcript antisense RNA (HOTAIR) detectable in body fluids, demonstrated their effectiveness in distinguishing RA patients from healthy individuals. This finding highlights the potential of exosomes as a promising alternative diagnostic platform for RA [65].

### Carbon-based nanoparticles

Certain autoimmune diseases may develop early in life, necessitating the use of highly sensitive, specific, and reliable biosensors to monitor disease progression. In addition to the previously discussed metal- and lipid-based systems, carbon-based NPs show strong potential for this purpose. Owing to their diverse structures and morphologies—including carbon quantum dots, fullerenes, and graphene—these NPs can be integrated into various biosensing platforms to enhance diagnostic accuracy and reliability. Carbon-based NPs possess properties such as good electrical conductivity, intrinsic fluorescence and high photostability which strengthen and amplify any signals detected by biosensors [66]. Other than their photochemical properties, carbon nanofiber electrodes with large surface area can also increase sensitivity and specificity of biosensors [67]. They have the ability to encapsulate more NPs and electrical conductivity to produce stronger electrocatalytic effect while maintaining cost-effectiveness to produce more reliable results in non-enzymatic glucose biosensors for the monitoring of blood glucose concentrations within T1D patients [67].

Furthermore, by using carbon-based NPs during imaging, this reduces the required contrasting agent dosage as carbon-based NPs have the capacity to produce better image contrast by strengthening X-ray signals at small dosage while minimizing the potential side effects brought by high dosage [68]. Besides typical imaging diagnostic tools such as MRI, CT and photoacoustic imaging, fluorescence imaging emerges as the next potential diagnostic tool as it offers effective diagnostic and prognosis of disease via observation of patients’ molecular pathology at high levels of sensitivity [68]. Carbon dots, exhibit extraordinary properties which enables it to enhance emission wavelength via the cleavage of specific C = N bond to stimulate the probe for positive signals and hence, improving diagnosis for RA patients [68].

Convenience is an important factor for patients with T1D, who must regularly monitor their blood glucose levels to prevent acute episodes and complications such as diabetic ketoacidosis. While numerous glucose sensors are currently available, most rely on invasive methods that penetrate the skin for measurement. Carbon-based NPs offer a promising alternative, enabling the development of highly sensitive and reliable wearable devices capable of real-time, non-invasive blood glucose monitoring to support more effective treatment planning. Carbon black, a type of carbon-based NP typically involved in wearable devices for glucose monitoring, contributes to high sensitivity via stronger electrical conductivity, cheaper production and ease of mass production [69]. This advancement could potentially encourage T1D patients to monitor their blood glucose levels more consistently, offering a convenient and affordable means of real-time, continuous monitoring that promotes better disease management.

## Nanoparticles for treatment of autoimmune diseases

Current therapeutic approaches for autoimmune diseases, primarily non-specific immunosuppressive agents and anti-inflammatory drugs, remain limited as they mainly function by suppressing excessive immune activity to reduce inflammation and relieve severe, painful clinical symptoms [37]. These immunosuppressive therapies provide only temporary disruption of the pathological pathways, necessitating long-term and consistent administration by patients [37]. Prolonged use may lead to the accumulation of cytotoxic drugs, increasing the risk of cancer and infections by suppressing the body’s normal immune functions needed to prevent disease flare-ups [37, 70–72]. Although more targeted therapeutic options are available, such as FDA-approved monoclonal antibodies (mAbs) that inhibit inflammatory pathways by targeting soluble inflammatory factors, limitations still exist [73]. Routine administration of mAbs, alongside non-specific immunosuppressants like corticosteroids and methotrexate, not only reduces pathological inflammation but can also impair normal physiological inflammatory responses. Despite their higher specificity, mAbs may inhibit critical cellular or cell–cytokine interactions, which can be detrimental when the body faces bacterial, viral, allergic, or oncogenic challenges [73].

Immune system dysregulation can contribute to both cancer and autoimmune disease, as the early stages of both conditions are promoted under similar pro-inflammatory environments characterized by reduced regulatory T (Treg) cell activity. However, as cancer progresses, its immunological profile diverges, placing it at an immunological opposite to autoimmune disease [74]. Patients with autoimmune diseases experience persistent Treg cell activity due to impaired Treg function, which promotes excessive production of pro-inflammatory cells and loss of self-tolerance. This dysregulation causes the immune system to attack the body’s own tissues, sustaining a chronic pro-inflammatory state. A pro-inflammatory environment then induces chronic inflammation, oxidative stress and DNA damage which promotes genomic instability and tumor initiation [29]. Conversely, tumor antigens can contribute to the development of autoimmune diseases through mechanisms such as epitope spreading or molecular mimicry, which trigger the production of autoantibodies. A well-documented example is idiopathic inflammatory myopathies (IIM), particularly the dermatomyositis (DM) subset, where factors including age, gender, and disease progression further elevate the risk of malignancy [29]. To address this challenge, dual-function NPs can be designed to elicit both pro- and anti-inflammatory responses depending on the stimulus, thereby enhancing the specificity of immune modulation and enabling the development of personalized treatment strategies [36]. NPs can be engineered to release their encapsulated molecules in response to specific stimuli, allowing them to remain inert while circulating in the bloodstream and only discharge their payload upon encountering particular physical or chemical triggers, such as changes in pH, temperature, ROS levels, enzymatic activity, or elevated adenosine triphosphate (ATP) concentrations [36].

Multiple similar immune mechanisms are involved in the pathogenesis of cancer and autoimmune diseases. Gut microbiome plays a critical role in the development of both cancer and autoimmune disease whereby composition of microbes and its interaction with our immune system may contribute to cancer tolerance, trigger anti-cancer responses or its involvement in the aetiopathogenesis of certain autoimmune disease [75]. The gut environment may be the potential answer to reduce flare-ups or even remodeling our immune system to re-establish the balance between self-tolerance and immune system to tackle autoimmune disease [75]. Interestingly, pathological studies of autoimmune disease contribute to the development of innovative anti-cancer drug as they have similar yet mirror-imaging molecular pathways [75].

A study revealed PTPN22 gene, a potential target for cancer immunotherapy and one of the players to inhibit antigen-specific responses of both T and B cells, with its Lyp620W variant found involved in multiple autoimmune diseases such as T1D, RA and SLE yet, it may lower cancer occurrence as carriers of PTPN22 (C1858T) variant have a reduced chance of non-melanoma skin cancer [75]. With such studies revealing their contradictory relationship, there is a need to strike a balance between both diseases to offer patients the best therapeutic method with the least consequential impact to ensure the best for their QoL for at least the next 5 years.

**Fig. 3:**
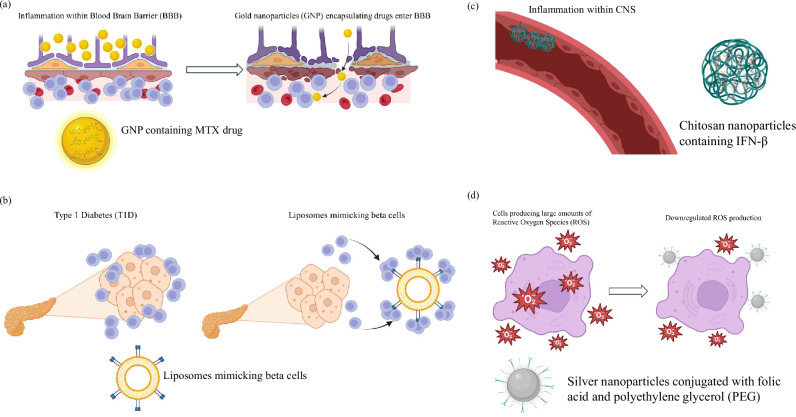
An overview of potential therapeutic methods to improve effectiveness of immunosuppressive treatments*.* **a** Gold nanoparticles, containing MTX drug, can pass through Blood Brain Barrier (BBB) to target inflammation within CNS. **b** Liposomes conjugated with protein-ligands to mimic beta-cells to increase self-tolerance and reduce immune cells from attacking beta-cells. **c** Chitosan nanoparticles containing IFN-β to specifically target inflammation within CNS. **d** Silver nanoparticles conjugated with folic acid and polyethylene glycerol (PEG) to reduce Reactive Oxygen Species (ROS) produced by cells to reduce possibility of cell death and inflammation.

The current clinical management of autoimmune diseases includes non-specific immunosuppressants, such as corticosteroids, and non-steroidal anti-inflammatory drugs (NSAIDs), which reduce inflammation by disrupting leukocyte function but are associated with undesirable side effects. While FDA-approved therapies can suppress resultant inflammation, they are unable to restore overall immune homeostasis [16, 73]. To restore immune homeostasis and control pathological inflammation, NPs can be used to target the upregulation of Treg cells [28]. Hence, NPs offer the potential to minimize or overcome the side effects of existing therapies, while preserving normal immune function and enhancing the therapeutic benefits of current immunosuppressive treatments (Fig. 3).

NPs possess inherent advantages over conventional therapies due to their small size, which allows them to traverse biological barriers, such as the BBB, enabling targeted drug delivery. This increases drug specificity, minimizes adverse effects, and helps overcome mechanisms associated with multidrug resistance [15]. Additionally, NPs can protect encapsulated drugs from enzymatic degradation and adverse microenvironments during circulation, thereby extending drug half-life and allowing for controlled, sustained release [15]. They also facilitate the delivery of poorly water-soluble drugs, potentially reducing required dosages while enhancing therapeutic efficacy without damaging healthy cells. Collectively, these properties improve the bioavailability and pharmacokinetics of therapeutics [15]. Beyond drug delivery, NPs with immunomodulatory functions can promote immune tolerance, prolong remission, and reduce flare-ups in autoimmune diseases by mechanisms such as downregulating ROS levels, delivering immunosuppressive agents to specific sites while evading the immune system, and exerting anti-inflammatory effects [76]. These mechanisms highlight the multifaceted therapeutic potential of NPs.

### Metallic nanoparticles

#### Gold nanoparticles (GNPs)

Beyond serving as drug carriers, NPs may have the potential to act as primary therapeutic agents. Modifications to their surface coatings can create diverse therapeutic properties, including anti-inflammatory, antimicrobial, and antioxidant effects, thus enabling immunomodulation that controls pathological immune responses, reduces drug dependency, and promotes immune self-tolerance. For example, an experiment conjugated R-dihydrolipoic acid (R-DHLA), known for its anti-inflammatory and oxidative stress–inhibiting properties, and cerium (Ce) to gold nanoclusters (AuNCs) to form R-DHLA-AuNCs-Ce, which demonstrated superior efficacy over methotrexate (MTX), the first-line treatment for RA, in experimental evaluations [55]. GNPs further exhibit anti-inflammatory and antioxidant effects through modulation of mitogen-activated protein kinase (MAPK) and nuclear factor kappa-light-chain-enhancer of activated B cells (NF-κB) signaling pathways. These effects include the reduction of pro-inflammatory cytokines, inhibition of ROS and nitric oxide (NO) generation, and downregulation of oxidative tissue damage markers [16]. Studies suggest that GNPs interfere with NF-κB signaling by preventing DNA binding affinity, inhibiting tissue plasminogen activator (TPA)-induced nuclear translocation, and disrupting thioredoxin reductase (TrxR) function, thereby downregulating TNF-α–induced NF-κB–dependent gene expression [16]. Experimental evidence supports this mechanism, showing that GNPs modify cysteine-179 of the IκKβ subunit, preventing NF-κB release and reducing the production of pro-inflammatory mediators across multiple cell types [16]. Moreover, GNPs possess the capability to influence macrophage polarization, particularly upregulating M2 (anti-inflammatory), due to the mononuclear phagocyte system (MPS) that enables macrophages to phagocyte GNPs, achieving macrophage-targeting to trigger generation of anti-inflammatory cytokines such as IL-10 and transforming growth beta (TGF-β) while lowering the production of pro-inflammatory cytokines such as IL-6, TNF-α, and IL-1β for faster anti-inflammatory effect to maximize therapeutic outcomes [77]. The inhibition of pro-inflammatory cytokines can also be achieved through cytokine-based therapies; however, their inherently short half-life significantly limits therapeutic efficacy. This limitation presents an opportunity for GNPs to exert anti-inflammatory effects, leveraging their inherent physicochemical stability and favorable biological properties to enhance treatment outcomes [78].

In addition, GNPs enhance BBB permeability by inhibiting the formation of specialized tight junctions (TJs) through suppression of the PKCζ isozyme, a key enzyme required for phosphorylation of zonula occludens-1 and occludin proteins that maintain BBB selectivity. This property facilitates the delivery of drugs to the CNS, aiding the treatment of CNS-related autoimmune diseases by reducing neuroinflammation [79]. Leveraging their inherent anti-inflammatory activity, GNPs are widely used to carry and deliver MTX to target sites under varying pH conditions, offering high drug-loading capacity and sustained release [80]. Moreover, GNPs can be synthesized using eco-friendly methods, such as metal–organic frameworks (MOFs), to maximize therapeutic efficacy while minimizing toxic side effects, including hepatotoxicity [80].

#### Silver nanoparticles (AgNPs)

Radical oxidative stress, an imbalance between free radicals and antioxidants, might be one of the factors leading to oxidative damage to DNA within cells and tissues [81]. This process upregulates superoxide anion radicals at sites of inflammation, perpetuating chronic inflammation and increasing the risk of gene mutations and cumulative DNA damage, which in turn elevates susceptibility to autoimmune diseases such as SLE, RA, MS and T1D [81, 82]. Increasing evidence highlights the unreplaceable role of oxidative stress in inducing tissue injury and immune activation, causing escalation in inflammatory responses which contributes to the vicious pathogenic cycle [83]. ROS play a pivotal role in the pathogenesis of RA, as excessive ROS production promotes lipid peroxidation, disrupts the calcium-to-phosphorus balance, impairs bone resorption, and delays bone repair through inflammatory upregulation. These processes contribute to complications such as bone fractures and prolonged pain in RA patients [82]. Interestingly, the pathological increase in vascular permeability observed in RA facilitates passive accumulation of nanocarriers via the Extravasation through Leaky Vasculature and subsequent Inflammatory cell-mediated Sequestration (ELVIS) effect, thereby enhancing NP-mediated drug delivery at inflammatory sites while minimizing systemic side effects. To address ROS-related autoimmune diseases, AgNPs represent a promising therapeutic candidate [84].

While AgNPs can generate ROS within cancer cells to induce cell apoptosis, they can also be ROS-scavengers [84–86]. The key regulating switch to differentiate AgNPs into an oxidant or antioxidant depends on the materials and concentration used to prepare AgNPs [87]. Antioxidants possess the ability to inhibit or slow down cell damage caused by oxidants such as ROS, reactive nitrogen species (RNS) or other unstable molecules by reacting with them at low concentrations yet sufficiently high to inactivate the target molecule and release a final product of lower toxicity than the eliminated radicals [87]. There is no all-round antioxidant to counter every radical due to the varying mechanisms required by different oxidants, giving rise to the need to find the required concentration of the correct antioxidant to achieve protection of the target molecule against that oxidant at a particular location within the body [87]. For AgNPs to achieve antioxidant properties, chemical preparation plays a significant role. An experiment involved prepared AgNPs with walnut green husk extract and compared the antioxidant ability of walnut (*Juglans regia*) extract to the prepared AgNPs via DPPH, a method used to measure antioxidant activity [87]. This revealed that AgNPs had stronger radical scavenging properties [87]. However, another experiment’s results showed contrasting evidence, in which the antioxidant capabilities of thirty plant extracts were stronger than increasing concentrations of Hi-media prepared AgNPs [87]. Preparation of AgNPs with polyphenols, phytochemicals and terpenoids from plants allows AgNPs to absorb these bioactive compounds, enabling them to perform singlet oxygen quenching and hydrogen donating, and to become a reducing agent, giving rise to their improved antioxidant properties [87]. Yet, many studies produce contradictory results, with some observing that AgNPs possess higher antioxidant properties while others observed the opposite. A possible explanation lies in the chemical reagents used to prepare AgNPs [87]. Another plausible reason for these fluctuating results is the lack of standard control, such as ascorbic acid, which would offer a more reliable comparison. As the aforementioned experiments conducted a relative comparison between their prepared AgNPs and the plant extract instead of a standard control, they concluded that their prepared AgNPs displayed a strong antioxidant effect [87]. This can potentially lead to biased conclusions. However, this can be overcome by establishing comparisons across multiple standard antioxidants against the prepared AgNPs to offer a more reliable outcome and reduce variability between experimental results of different studies.

AgNPs can be synthesized by physical, chemical or biosynthetic methods. Green synthesis is highly favorable as it lowers nanotoxicity by utilizing natural ingredients and offers a more sustainable production by reducing the potential detrimental environmental impacts [88]. The toxicity of AgNPs originates from their pro-oxidant capabilities, which can be controlled by the method of synthesis. An experiment conducted an in vivo comparison between chemically synthesized AgNPs (C-AgNPs) and green synthesized AgNPs (G-AgNPs), which were monitored by comparing biological markers and the histopathology of various organs [88]. The endogenous antioxidant enzymes monitored were Catalase (CAT), superoxide dismutase (SOD) and glutathione-*S*-transferase (GST) [88]. G-AgNPs induced a mild increase in CAT, SOD and GST levels, indicating that G-AgNPs have a stronger antioxidant effect than C-AgNPs, as the increased GST levels suggested increased activity of antioxidant enzymes for ROS removal [88]. Although the method of production affects nanotoxicity, other factors such as particle size, shape and concentration are equally important. Despite synthesizing AgNPs in a green methodology, they still exhibit certain toxicity, yet this is minimal when compared to cadmium metal and C-AgNPs, based on observed damage to kidney, liver and intestine tissues [88]. Therefore, more research is required to maximize the antioxidant effects of AgNPs to minimize their potential detrimental impact on patients’ health.

In addition to chemical preparation and method of synthesis, concentration is another factor to consider. An experiment evaluated the antioxidant ability of increasing concentrations of coconut meat-AgNPs (Cm-AgNPs), ranging from 25 to 300 μg/ml in increments of 50 [89]. The results demonstrated a linear relationship between Cm-AgNPs concentrations and antioxidant capabilities, with the highest concentration of Cm-AgNPs serving as the strongest radical scavenger [89]. This revealed that the antioxidant capabilities of AgNPs are highly dependent on their concentration [89]. At higher concentrations, these bio-derived AgNPs exhibit remarkable antioxidant activity, contributing to their therapeutic value in mitigating oxidative stress-related conditions [87]. An experiment utilizing AgNPs conjugated with folic acid (FA) and polyethylene glycol (PEG), forming FA-AgNPs, was conducted in a RA mouse model to induce apoptosis of pro-inflammatory M1 macrophages and promote M2 macrophage polarization. The results demonstrated a significant reduction in ROS levels within activated macrophages treated with either AgNPs or FA-AgNPs, confirming the strong ROS-scavenging capability of AgNPs [84]. However, the findings indicated that AgNPs exhibited approximately threefold higher toxicity compared to FA-AgNPs, highlighting the need for innovative strategies to mitigate the cytotoxic effects of AgNPs while preserving their ROS-reducing and anti-inflammatory benefits. After 28 days of treatment, evaluations of serum biochemical and hematological parameters were performed, yielding favorable results. All indicators remained within normal ranges, and no signs of hepatic or renal damage were observed. These findings confirm that FA-AgNPs can be safely eliminated from the body without causing long-term toxicity during or after the 28-day experimental period. An experiment comparing the superoxide scavenging abilities of three types of NPs, GNPs, AgNPs, and platinum (PtNPs), revealed that AgNPs exhibited the strongest superoxide inhibition activity, followed by PtNPs and then GNPs [90]. Although higher AgNPs concentration contributes to a strong reducing ability, whereby more available AgNPs can reduce more ferric ions into ferrous ions [91]. This exhibits stronger antioxidizing ability and demonstrates a dose-dependent antioxidant effect [91]. However, as concentration increases, the risk of adverse health complications arises, which requires further research to strike a balance between concentration and nano safety.

With these demonstrated advantages, AgNPs have shown strong potential as therapeutic agents, functioning either as drug carriers or as immunomodulatory particles that promote the reestablishment of self-tolerance. Their well-characterized mechanisms of action and ability to reduce patients’ susceptibility to infections further underscore their promise in therapeutic applications.

### Polymer-based nanoparticles

Polymer-based NPs, composed of repeating monomeric units that confer structural versatility for diverse applications, can be derived from either synthetic or natural sources. These polymers offer high biocompatibility and biodegradability while exhibiting markedly reduced levels of nanotoxicity. Synthetic polyester-based NPs are one of the most universally used materials due to their high stability and ease of synthesis, making up 29% of FDA-approved NP drugs [92, 93]. Although immunosuppressive drugs may inhibit progression of autoimmune diseases, it should not be the long-term solution as it can potentially increase fracturing risk, especially at the hips for patients with autoimmune disease, which requires caregivers to be extra cautious to minimise any underlying risk of falling [94], infection and malignancy [95]. Inducing immune tolerance presents a more effective therapeutic strategy by specifically targeting APCs, particularly DCs, which play a pivotal role in regulating T-cell activation and tolerance. When DCs activate Tregs, they promote the expansion of Tregs while suppressing T-cell proliferation, thereby re-establishing immune tolerance [96]. Thus, with multiple polymer-based NPs, this enables us to either deliver specific biologics or potentially achieve re-establishment of immune tolerance which will be discussed in the following sections.

#### Chitosan-based nanoparticles

Interferon Beta (IFN-β) is a type 1 interferon involved in reducing inflammation, and is currently used as a first-line systematic treatment for MS to reduce loss of motility and downregulate inflammation [97]. Administration of IFN-β, typically via intramuscular or subcutaneous injection, has been shown to reduce relapse frequency, attenuate CNS inflammatory damage, and slow disease progression in approximately 30% of patients [97]. IFN-β is often administered in high doses and frequencies due to its short half-life and inaccessibility to the CNS due to the presence of the BBB [97]. With such high dosages and frequencies, this can lead to the secretion of IFN-β neutralizing antibodies, further reducing IFN-β bioavailability and clinical efficacy [97]. In addition to the requirement for high doses of IFN-β, frequent injections can cause local complications such as infection, inflammation, and erythema, thereby intensifying patient discomfort. These adverse effects make treatment adherence challenging, hindering effective management of MS and increasing the likelihood of suboptimal therapeutic outcomes [97].

To resolve this challenge, chitosan-based NPs can be used to encapsulate IFN-β and transport it to the site of inflammation for regulation of autoimmune disease. Chitosan, one of the most naturally abundant biopolymers, is a cationic polysaccharide extracted via deacetylation of chitin obtained from outer shells of crustaceans, which is often involved in wound recovery, cytokine production and activation of cell polymorphism with mucosal adhesion properties [98, 99]. These characteristics confer chitosan-based NPs with advantageous properties, including non-toxicity, high biocompatibility, and biodegradability, leading to chitosan being designated as “Generally Recognized As Safe” (GRAS) by the FDA [99, 100].

Although extensive research has focused on elucidating the pathology of MS, its precise underlying mechanisms remain unclear. Nevertheless, since most MS patients exhibit CNS-related clinical symptoms, the CNS has become the primary target for therapeutic interventions aimed at slowing disease progression, prolonging remission, and reducing acute relapses [101]. Drug delivery to the CNS is particularly challenging due to the highly selective BBB, which permits passage primarily of small hydrophobic molecules. Systemic administration of drugs can result in off-target exposure to healthy cells, causing undesirable side effects. Consequently, newly developed CNS-targeted therapies often exhibit higher failure rates compared to drugs aimed at non-CNS tissues [102]. With controlled drug delivery of IFN-β via intranasal administration pathway by chitosan NPs, this minimizes possible drug exposure and interaction with normal healthy cells [97]. This decreases unfavorable side effects and offers a more localized drug delivery for better regulation of neuroinflammatory process [97]. Furthermore, chitosan NPs protect IFN-β from enzymatic degradation and offers consistent prolonged release of cytokine, increasing half-life and bioavailability of IFN-β [97]. This approach could potentially reduce both the frequency and dosage of IFN-β administration. Previous studies have demonstrated that intranasal delivery results in more efficient cytokine accumulation in the CNS of both healthy rats and non-human primates [97].

Chitosan NP is a good choice for intranasal drug delivery given its cationic nature. It offers strong adhesive ability to the anionic sialic acid present on mucosal surface which increases drug bioavailability in an extensively blood-capillaries rich environment within the nasal cavity, enabling higher drug absorption [97]. Chitosan NP also encourages TJ permeability in nasal epithelium through opening these TJ temporarily and hence, upregulating paracellular transport of drug across cell membrane barriers [97]. Experiments have shown that intranasal administration of chitosan NPs encapsulating IFN-β achieves remarkable therapeutic efficacy, allowing a 50% reduction in IFN-β dosage and a 33% decrease in treatment frequency [97]. This equates to decrease in a total of 78% of original daily therapeutic amount, from normal daily requirement of free 200,000 IU IFN-β to 50,000 IU IFN-β to achieve the same therapeutic effect [97].

Furthermore, intranasal administration of chitosan NPs is nontoxic and gentle to nasal mucosa, exhibiting neuroprotective function which potentially downregulates neuroinflammation, thus slowing disease progression [97]. Most importantly, chitosan NPs exhibit minimal side effects, as they do not induce hemolysis and are unlikely to cause renal toxicity, even at high concentrations [100]. Furthermore, chitosan NPs encapsulating siRNA or DNA have shown success in downregulating inflammation via limiting the generation of pro-inflammatory mediators or silencing CCR2 expression in arthritic rats via its cationic nature [103]. Given their widespread use in applications such as food packaging, antioxidant and antibacterial research, and drug delivery, chitosan NPs’ extensive utilization attests to their low nanotoxicity. Moreover, their cationic nature provides opportunities for surface modification with diverse materials, allowing optimization of their properties to maximize therapeutic efficacy. In addition to naturally occurring polymers, synthetic polymers (particularly PLGA and PLA NPs derived from renewable sources) can be employed to meet broader therapeutic demands [98].

#### PLGA/PLA-based nanoparticles (PLGA NPs)

Polylactic acid (PLA) and polylactide-co-glycolide (PLGA), both synthetic polymers, are highly biocompatible and biodegradable, earning approval from the European Medicines Agency (EMA) and the FDA. Upon degradation within the body, PLGA breaks down into its monomeric components, glycolic acid and lactic acid, which are naturally metabolized via the Krebs cycle. This efficient biotransformation process minimizes residual toxicity, making PLGA an ideal and safe candidate for use in drug delivery systems [104]. These properties have facilitated their widespread use in various biomedical applications [105]. When these PLA or PLGA-based NPs are conjugated with cyclosporine A or myelin, they become highly competent to induce T cell tolerance [103]. This reduces recurrence and delays disease progression in mouse models with EAE and MS [103]. Other than being highly biocompatible and biodegradable, it possesses multiple advantages such as increased protection of biomolecules encapsulated, decreased dose frequency and faster degradation while maintaining sustained release of drugs [98].

To prevent rapid metabolic clearance of drugs and NP aggregation during prolonged circulation, a study employed a PLGA NP delivery system with sustained-release properties in vitro. This system effectively encapsulated highly hydrophobic natural compounds, resveratrol (Res) and betulinic acid (BA), enhancing their anti-inflammatory effects and yielding significantly improved therapeutic outcomes in ulcerative colitis (UC) [80]. However, passive targeting, where drugs are delivered to a target site without precise control over release, may be suboptimal. This limitation has led to the development of active targeting strategies, which employ ligand conjugation to achieve highly specific and selective binding to cells based on the receptors involved in various autoimmune diseases [80]. Furthermore, PLGA NPs exhibit exceptional stability in encapsulating biologics, enabling new therapeutic strategies. For example, small-interfering RNA (siRNA) technology offers high specificity in silencing disease-causing genes, but its clinical potential is limited by poor bioavailability, rapid clearance, and restricted tissue penetration, challenges that can be effectively addressed using NP-based delivery systems [106]. PLGA NPs overcome these limitations by physically shielding siRNA within a hydrophobic polymer matrix that protects it from nuclease degradation, prolonging systemic circulation through reduced renal filtration, and enhancing cellular uptake via endocytic pathways [107, 108]. In addition, the tunable lactic/glycolic acid ratio of PLGA enables controlled degradation and sustained release, maintaining therapeutic siRNA levels over longer durations [109]. A study revealed success involving delivery of CCR2 short hair-pin (shRNA)-loaded EGFP-EGF1-conjugated PLGA NPs (ENPs) to silence CCR2 expression in atherosclerotic cellular models of macrophages, regulating inflammation related to macrophage [76]. Although highly biocompatible, addressing nanotoxicity remains a critical concern. A study developed a monomethoxy-(polyethylene glycol)–poly(*D*, *L*-lactide-co-glycolide)–poly(*L*-lysine) (mPEG–PLGA–PLL) NP delivery system to transport microRNA-125a (miR-125a) to splenic T cells, termed miR-125a-loaded mPEG–PLGA–PLL (PEAL_(miR-125a)) NPs, for therapeutic intervention in SLE. The study compared these NPs with direct administration of dexamethasone (Dex) to evaluate their respective effects on T-cell toxicity. PEAL_(miR-125a) NPs exhibited minimal impact on T-cell apoptosis and proliferation while effectively impeding SLE progression, whereas Dex induced significant T-cell apoptosis and inhibited proliferation. The NPs protected miR-125a from degradation, prolonged its circulation in vivo due to PEG-mediated steric stabilization, and facilitated preferential accumulation in the SLE-affected spleen through size-dependent biodistribution [110–112]. This targeted accumulation enhanced specificity toward pathological splenic T cells, minimized off-target effects, and maximized therapeutic efficacy with negligible toxicity [80]. A study involving PLGA NPs carrying autoantigens, myelin oligodendrocyte glycoprotein (MOG_35-50_) and Interleukin-10 (IL-10) instead of immunosuppressive drugs, has been observed to reduce histopathological damage of central nervous tissue and decrease secretion of IL-17 and IFN-γ, moderating the development of EAE [96]. This shows the high specificity and stability of PLGA NPs as a delivery carrier to achieve potential therapeutic outcomes. Furthermore, PLGA NPs support prolonged antigens release and immune regulatory cytokines, aiding in Treg induction, and retaining the capability to induce both B and T-cell tolerance to induce immune tolerance [95]. This establishes a foundation for immunotherapy in which NPs can potentially replace conventional drugs, achieving more sustained immunomodulatory effects. Given their safety profile, biocompatibility, and versatility in drug delivery, PLGA NPs have emerged as one of the most widely used and effective polymer-based delivery systems [98].

### Lipid-based nanoparticles

#### Liposomes

In addition to alleviating pain, liposomes can function as immunotherapeutic agents by mimicking apoptotic β-cells to restore self-tolerance in T1D, potentially reducing reliance on insulin injections and improving patient quality of life. Beyond T1D, in vivo studies using phosphatidylserine liposomes loaded with myelin-oligodendrocyte glycoprotein peptide 40–55 (MOG) for the treatment of mice with experimental EAE, a model of MS, demonstrated favorable outcomes by reducing both the incidence and severity of disease [113]. Given the small size and hydrophobic nature of liposomes, this enables liposomes to pass through the BBB to deliver drugs targeting the CNS for MS treatment. In mice with proteolipid protein (PLP)-induced EAE, treatment with glucocorticoids encapsulated in nano-sterically stabilized liposomes (nSSL) resulted in faster recovery from acute disease episodes compared to mice receiving conventional MS therapies, such as Betaferon and Glatiramer Acetate (GA, Copaxone) [114]. Liposomes possess hydrophilic properties with hydrophobic core, giving them the ability to carry both hydrophilic and hydrophobic molecules effectively [98]. Furthermore, the extensive application of liposomes in food packaging and preservation has highlighted their superior encapsulation capabilities. Studies have shown that liposome-coated cheese maintained significantly lower bacterial counts over a 4-week storage period compared to both uncoated and chitosan-coated cheese, demonstrating their effectiveness in preserving bioactive compounds [98].

In addition to facilitating targeted drug delivery to sites of infection, NPs can also enable the stable delivery of RNA interference (RNAi) therapeutics. This approach involves administering small interfering RNA (siRNA) to induce gene silencing, thereby downregulating genes implicated in the pathogenic pathways of autoimmune diseases and offering a promising mechanism for immunomodulation [115]. Studies have been conducted to study siRNA, and development of multiple siRNA delivery systems have shown the importance of siRNA in gene silencing [115]. However, purely introducing siRNAs into patients might result in poor treatment efficacy [115]. siRNA therapeutics face several challenges, including low cellular uptake, rapid degradation by nucleases and plasma enzymes, extracellular barriers such as immune system clearance, and intracellular obstacles like inefficient endosomal escape and off-target effects. These limitations result in premature degradation before reaching target cells, ultimately shortening the effective half-life of siRNAs [115]. Liposomes can encapsulate siRNA to prevent degradation while transporting it to target site, thus maximizing treatment efficacy and enhancing siRNA’s half-life. There are two types of liposomes, cationic and neutral liposomes, that forms lipoplexes when containing siRNA [115]. Cationic liposomes are preferred over neutral liposomes as they have shown outstanding performance in delivery of siRNAs [115], making cationic property an important characteristic as the highly selective BBB is resistant against anionic charges [79].

Although studies have shown that cationic NPs exhibit higher toxicity than anionic NPs, with experimental results indicating greater neuronal loss in the brains of rats treated with cationic NPs, their therapeutic advantages often outweigh these limitations [79]. These limitations can be easily overcome by coating the surface of cationic lipoplexes with harmless and biodegradable anionic polymers which upregulates in vivo siRNA delivery in tissues of lungs and liver while minimizing toxicity [115]. In 2020, ONPATTRO^®^ (patisiran), a lipid NP containing siRNA, was recognized as both the first targeted siRNA-lipid NP mediated RNA-based gene therapy and the first FDA-approved treatment drug for the treatment of polyneuropathy caused by hereditary transthyretin-mediated amyloidosis, a type of inherited genetic disorder [115–117]. This advancement showcases the potential of using siRNA-lipid NPs for targeted therapies to control pathogenic inflammation in autoimmune diseases.

Furthermore, liposomes are a better option as they offer high biocompatibility, biodegradability and low toxicity as compared to synthesized polyethyleneimine (PEI). PEI polymer NPs is the gold standard for gene transfer given its high competency in siRNA transfection, yet its high cytotoxicity remains as a challenge even after modification [115]. Modified liposomes conjugated with PEI-PEG, referred to as PEI-PEG polyplexes, were used in an experiment to enhance gene-silencing efficiency, achieving up to 42% suppression of the EGFP gene [115]. However, PEI-PEG polyplexes also induced undesirable pro-inflammatory effects, evidenced by increased cytokine production, highlighting the need for further modifications to mitigate this limitation.

Moreover, chitosan-lipid NPs encapsulating indomethacin helps to prevent direct exposure of indomethacin to the gastrointestinal tract, increasing drug stability as indomethacin has detrimental impact to the gastrointestinal tract [80]. Since liposomes encapsulate drugs within their lumens, this reduces off-target implications while enhancing therapeutic effect, which was proven by an experiment that compares the therapeutic effect between liposome-free Dexamethasone (DEX) and DEX-loaded liposomes [118]. Both were loaded into RA mice models respectively and DEX-loaded liposomes displayed better anti-arthritogenic properties than liposome-free DEX [118]. Furthermore, the delivery of weekly intraperitoneal injections with mycophenolic acid (MPA)-loaded liposome nanogels regressed the onset of kidney disease and extended the life-span for mice models with disease [118], proving the efficacy of liposomes to deliver drugs without causing harmful side effects.

Modifications such as coating the liposome bilayer with amphiphilic PEG extend circulation time by preventing opsonization, allowing the particles to evade the reticuloendothelial system (RES) and the immune system, thereby prolonging their presence in the bloodstream [119]. Modified liposomes with extended circulation time, referred to as long-circulating liposomes, improve drug-loading efficiency and enhance accumulation at target sites through high-affinity binding, thereby increasing the likelihood of successful treatment in patients with RA and MS [103]. Liposomes have the capacity to contain many different types of drug such as dexamethasone (Dex), prednisolone (PRD), methotrexate (MTX), glucocorticoid and FK506 to achieve superior therapeutic success in-vivo and in-vitro [103].

#### Exosomes

Exosomes are a subtype of extracellular vesicles secreted by virtually all cell types into the extracellular space. They exhibit high biocompatibility and stability, possess immunomodulatory activity, and can traverse selective biological membranes, making them versatile for multiple biomedical applications [80]. Because most cells produce exosomes, their molecular cargo can reflect an individual’s current physiological or pathological state, and exosomes can be readily isolated from common body fluids (e.g., semen, saliva, urine, tears, and synovial fluid) [119]. Moreover, exosome surface markers can provide clues to their cellular origin. For example, tumor-derived exosomes are often abundant and display disease-associated biomarkers on their surface [120, 121]. This principle supports the potential use of exosomes as non-invasive diagnostic tools for organ-related diseases, including kidney and genitourinary tract conditions, where urine-derived exosomes can be collected and analyzed [120].

Beyond diagnostics, exosomes play key roles in cell–cell communication through surface receptors and ligands that mediate interactions with recipient cells [119]. This enables exosomes to deliver bioactive molecules from parental to target cells [119], supporting their use as therapeutic delivery vehicles in autoimmune disease settings. In an experimental autoimmune encephalomyelitis (EAE) model, bone marrow mesenchymal stem cell (BM-MSC)–derived exosomes promoted microglial polarization toward an M2 phenotype, reduced pro-inflammatory mediators (e.g., TNF-α and inducible nitric oxide synthase, iNOS), and increased anti-inflammatory mediators (e.g., IL-10, TGF-β, and arginase-1), collectively reducing neuroinflammation [120]. MSC-derived exosomes can also suppress pro-inflammatory cytokine gene expression and reduce Th1/Th17 differentiation, thereby lowering inflammatory mediator production [120]. Their appeal is further strengthened by their dual regulation of innate and adaptive immunity: they promote anti-inflammatory M2 macrophage polarization while suppressing T-cell proliferation and supporting regulatory T-cell (Treg) induction, fostering immune tolerance [122]. Mechanistically, exosomal miRNAs (e.g., miR-223 and miR-146a) can reprogram macrophage function by acting on important signaling nodes such as NF-κB, signal transducer and activator of transcription (STAT), and phosphatidylinositol 3-kinase/protein kinase B [122]. In addition, MSC-derived exosomes may support tissue repair by preventing excessive inflammation and helping restore immune homeostasis [122].

Because exosomes are naturally released into extracellular fluids, they can be collected from patients and engineered to carry specific agents or drugs within their lumen for autoimmune disease diagnosis and therapy [119]. Importantly, their endogenous membrane proteins and ligands generally confer low immunogenicity, reducing the risk of unfavorable immune activation [119]. Exosomes also share key membrane and cargo features with their parental cells [123], contributing to their capacity to influence neighboring cells, distant tissues, and local microenvironments [123]. These factors can potentially induce systemic effects such as preventing immune responses through the expression of highly specific exosome membrane proteins while maintaining low immunogenicity [123]. The membrane protein compositions of exosomes are influenced by pathological conditions [124]. Notably, pathological conditions can reshape exosomal composition. Genetic or disease-associated perturbations can alter exosomal trafficking and fusion machinery, including RAS-associated binding guanosine triphosphate (RAB GTPases), cytoskeletal proteins and soluble N-ethylmaleimide-sensitive fusion attachment protein receptor (SNARE) on exosomes. These observations have motivated the development of engineered exosomes to modulate cellular microenvironments and physiological states, with the aim of delaying disease progression or reducing recurrence [124]. Their cellular origin also facilitates membrane fusion and cargo release into recipient cells, enabling drug delivery to sites that are otherwise difficult to access, including the CNS [119].

For example, exosomes derived from RAW macrophages and loaded with resveratrol reportedly reduced inflammatory responses in both central and peripheral nervous systems in an MS mouse model [119]. Intranasal administration slowed disease progression and reduced severity, with histopathology indicating reduced inflammatory mediators in brain, spinal cord, peripheral nervous system, blood, and spleen, alongside improved myelin recovery [119]. These effects may reflect both the delivered drug and anti-inflammatory RNAs intrinsic to exosomes [119]. Because exosomes from different cell types carry distinct surface markers and protein profiles, they may enable relatively targeted therapeutic effects across autoimmune contexts with limited additional modification. In another example, MSC-derived exosomes carrying miRNA-216a-5p (implicated in spinal cord recovery) promoted repair in models of traumatic spinal cord injury, underscoring the potential of exosomes as functional cargo carriers [119]. More broadly, patient-derived exosomes, whose cargo reflects individual health states, raise the possibility of personalized approaches, given the roles of exosomes in maintaining homeostasis and coordinating tissue responses [125]. Furthermore, exosomes can also mediate protective cell-cell interactions, for instance, astrocyte-derived exosomes have been reported to reduce neuroinflammation, increase antioxidant enzyme activity, lower oxidative stress, and improve functional recovery [119].

Clinical interest in exosomes has expanded substantially. Multiple studies and clinical trials across disease areas (including cancer, cardiovascular disease, and autoimmune conditions) have explored exosomes for diagnosis and therapy, with some evidence suggesting advantages compared with allogeneic MSC transplantation in certain contexts [119, 126, 127]. Beyond serving as drug carriers, MSC-derived exosomes may act as therapeutic agents by modulating both innate and adaptive immunity, for example, reducing pro-inflammatory cytokines (e.g., IL-2, TNF-α, IFN-γ) while promoting anti-inflammatory programs [120]. In regenerative applications relevant to autoimmune complications, human Wharton’s jelly MSC-derived exosomes contain diverse miRNAs associated with cartilage repair. Exosome incorporation into acellular tissue patches has been reported to promote hyaline cartilage regeneration in vitro and in vivo, supporting the concept of exosome-enabled repair strategies for RA-related tissue damage (e.g., erosion and degeneration) [128].

Exosomes also offer a potential alternative to conventional anti-inflammatory medications. Since inflammation is a core feature of autoimmune disease [120], anti-inflammatory drugs such as NSAIDs are frequently used but can cause gastrointestinal side effects [120]. Exosome-based delivery systems may help localize drug exposure and reduce off-target immune activation, potentially improving tolerability.

Exosome modification can be performed endogenously or exogenously. Endogenous loading incorporates therapeutic agents into parental cells (e.g., by drug incubation or delivery of drug-encoding DNA/RNA), resulting in secretion of drug-loaded exosomes [129]. This approach generally preserves exosome integrity while enabling therapeutic function [129]. For example, exosomes derived from MSCs pre-treated with paclitaxel showed stronger anti-proliferative effects than exosomes from untreated MSCs, supporting the feasibility of parental-cell preconditioning [129]. To minimize potential damage to the immunosensitive surface membrane of exosomes via exogenous loading, an experiment performed chemical ligation by fusing parental cells with membrane-fusion liposomes while engineering the exosomes [129]. This reduces the compromise of exosomes membrane integrity while enabling the encapsulation of drugs to perform their therapeutic function at the desired target site [129]. Other than the above-mentioned liposome ligation, modifying the surface of exosomes with other materials such as GNPs, which increases efficacy in drug delivery to the brain as GNPs exhibit extraordinary capabilities to pass through the highly selective BBB and bind to brain cells under laminar flow conditions [129]. Other than chemical ligation, methods such as sonication and electroporation are used yet, they vary in their damage to membrane integrity. Sonication deforms the exosome membrane to create transient small pores that allow small molecules to diffuse into exosomes, while electroporation creates temporary hydrophilic pore channels for small molecules to enter exosomal capsules [129]. Although both methods compromise membrane integrity, electroporation might be superior to sonication as the damage it causes is temporary. However, a challenge remains in using this technology which would be to increase stability of exosomes. Creating temporary hydrophilic pore channels increases exosomal instability, leading to aggregation of exosomes [129] before they can deliver drugs to target site. This suggests that these two methods might not be ideal and that more innovative ways of incorporating drugs into exosomes are required [129]. Consequently, the endogenous method is preferable to the exogenous method, as it does not compromise on membrane integrity. This maintains the specific arrangement of proteins on the surface of exosomes, which is essential for low immunogenicity during drug delivery to target sites.

Before delivering each unique, engineered exosomes, there is a need to observe whether the modified exosomes still retain the same membrane protein compositions as their parental cells. Labeling methodology, such as fluorescence, can be utilized to visualize them. However, such methodologies might not be ideal, as labeling may cause undesired changes to molecular structure of the exosomes, affecting the binding affinity between exosomes and target molecules [130]. Furthermore, evaluating the therapeutic efficacy of exosomes is highly dependent on monitoring disease progression by observing changes in pathological tissues [131]. It is difficult to detect the footprints of exosomes without any labeling after their introduction into patients, as it can be taken up by multiple cells and tissues [131]. This reveals a need for more research into the development of a tracking system to determine the specific mechanisms of exosomes and to maximize their therapeutic capacity [131]. Research has offered label-free detection techniques which can potentially monitor exosomes before and after administration without influencing the interaction between target molecules and exosomes. Label-free detection techniques include signal, optical or electrical-based transduction whereby electric-based, mainly field-effect transistor (FET) and electrical impedance spectroscopy (EIS) biosensor, have garnered attention due to their highly efficient and sensitive analysis of exosomes [132]. They capture the characteristics of membrane protein, without the need for any labels, via detection in electrical charge distribution when specific binding happens, lowering potential damage to membrane integrity since there is no involvement of pressure [132]. Lab-on-a-chip (LoCs) is another innovative technique that involves microfluidic technology, incorporating multi-step procedures such as purification, isolation, and analysis of exosome membrane composition [132]. This preserves the membrane integrity while working with minimal sample volumes and concentrations [132]. An experiment revealed that a lab-on-a-disc integrated with two nanofilters (Exodisc) is highly sensitive, removing more than 95% of protein contaminants to achieve excellent recovery efficiency [133]. Meanwhile, mRNA analysis showed concentrated mRNA of more than 100-fold higher than those obtained via ultracentrifugation [133]. Therefore, with further advancement in these technologies, it will be possible to analyze modified exosomes without compromising membrane integrity while ensuring nano safety.

The presence of exosomes in various cellular processes, such as immunomodulation, reprogramming and remodeling, and signaling pathways for both physiological and pathological conditions has demonstrated its importance [134]. In 2022, a review stated that there were 116 clinical trials involving exosomes, with 58 clinical trials using exosomes as a biomarker, 33 for exosome therapy and 6 for drug-delivery system [134]. A search of clinicaltrials.gov to date reveals an increasing number of clinical trials in using exosomes for therapeutic purposes. Using keywords “autoimmune” and “exosomes”, the search revealed 3 clinical trials with the status “not yet recruiting” and 1 clinical trial with the status “recruiting”, all of which involve treatments for SS to relieve dry eye syndrome. Additionally, 1 clinical trial with the status “active, not recruiting” aims for autoimmune encephalitis. Although the website listed 8 clinical trials involving exosomes for therapeutic purposes, only five have proceeded with their research while the remaining have an “unknown status” regarding their potential application for T1D and MS. Most clinical studies involves MSC-derived exosomes, highlighting their role in regenerative medicine, yet due to limited research, its unknown side effects have deterred their transition from bench side to bedside [134]. Therefore, further research, beginning with well-defined indications (e.g., symptomatic relief) and progressing toward disease-modifying endpoints, is needed to clarify mechanisms, optimize engineering approaches, and expand therapeutic applications of exosomes in autoimmune disease.

### Carbon-based nanoparticles

Carbon-based NPs have potential to advance autoimmune disease management beyond symptom control by enabling strategies that move toward disease modification and, potentially, curative outcomes. In T1D, insulin injections remain the standard approach for glycemic control because oral insulin is largely ineffective: enzymatic degradation in the gastrointestinal tract reduces bioactivity before insulin can reach target tissues. This limitation may be addressed using pH-responsive, carboxylate-terminated carbon-based nanomaterials engineered to protect insulin from degradation and improve delivery efficiency [67]. In one study, a significant reduction in blood glucose was reported within 42 days in vitro and in mouse models [67]. Beyond glycemic control, carbon nanomaterials have also been explored in injury models. For example, carbon NPs formed from hydrophilic clusters and conjugated with polyethylene glycol (PEG) were reported to improve outcomes including infarct size, hemispheric swelling, hemorrhage, Bederson score, and mortality when administered within 2 h of treatment in experimental models [67].

In addition to diagnostic and therapeutic applications, carbon-based NPs such as carbon nanotubes and C₆₀ fullerenes have demonstrated effects relevant to metabolic health. In obese mouse models, dietary incorporation of these NPs normalized body mass index and modulated cytokine release. This observation may be relevant for patients with autoimmune diseases, who can develop obesity-related complications associated with chronic inflammation, and could therefore benefit from interventions that improve metabolic status and QoL [67].

Carbon-based NPs also exhibit strong adsorption capacity for circulating compounds, including drugs, toxins, and metabolites [135]. This property may help reduce systemic toxicity and metabolic complications associated with some NP-based drug delivery systems [135]. Nanodiamonds, for example, have been reported to adsorb inflammatory cytokines, suggesting a possible approach to reduce flare frequency in autoimmune disease [135].

Finally, carbon-based materials may support management of metabolic and excretory complications in autoimmune conditions. In one experiment, a hemodialysis membrane composed of polyethersulfone and carboxyl-functionalized multi-walled carbon nanotubes showed high biocompatibility. The membrane reduced cholesterol (a risk factor for downstream complications), enhanced urea and creatinine clearance, and decreased membrane fouling by ~30%. These improvements support the feasibility of integrating carbon-based nanomaterials into extracorporeal or supportive platforms that improve clearance while maintaining material stability and performance [135].

## Current limitations of nanoparticles

In current clinical applications, only a limited number of high-quality excipients, such as specific lipids and human serum albumin, have been approved for use in NP formulations. Additionally, some NPs exert their therapeutic effects only upon interaction with cell surfaces, necessitating high biocompatibility to achieve optimal efficacy [136]. Despite the strengths of NPs discussed above, most NP systems remain in the experimental stage (Supplementary Table S1) because uncertainties and safety concerns persist when they are introduced in vivo [46]. Although many studies use animal models, physiological differences between these models and patients with autoimmune diseases limit direct translation. Future research should better replicate patient-specific physiological conditions, which may facilitate clinical translation and strengthen confidence in nanotechnology-based therapies [137].

The design and engineering of NPs also remain complex, because multiple interdependent factors such as size, shape, and material composition, affect both safety and efficacy. For example, larger NPs can carry higher drug loads, potentially reducing dosing frequency and improving therapeutic outcomes. However, increasing size may raise toxicity risk and the likelihood of anatomical complications, such as obstruction of narrow blood vessels, which can lead to serious events like pulmonary embolism [37]. Larger particles may also attract phagocytic immune cells, resulting in premature clearance before NPs reach the target site and thereby reducing treatment effectiveness [37]. However, smaller NPs are more prone to aggregation and may evade clearance by the reticuloendothelial system (RES), making complete elimination more difficult and increasing the risk of cumulative toxicity [104]. Optimizing particle size between 10 and 100 nm helps prevent splenic capture while remaining large enough to avoid rapid renal filtration, thereby minimizing the risk of damage to narrow capillaries within the kidneys [138].

Drug concentration at the target site is another critical constraint, and it varies with the route of administration. During transit to inflamed tissues, drug concentration can drop rapidly, which may necessitate a high initial dose. This can exceed toxicity thresholds yet still fail to achieve adequate exposure at the target site. This challenge motivates the use of NPs as drug carriers to enhance delivery efficiency and sustain local drug concentrations [139].

Coating choice is also central to balancing safety and performance. Appropriate coatings can reduce toxicity and improve stability, while enabling gradual payload release, targeted delivery, and potentially lowering the risk of drug resistance [139, 140]. For instance, incorporating AgNO₃ into the porous, networked polymeric coating along with surfactants and *L*-ascorbic acid, produced a low-cytotoxicity carrier that retained AgNPs without releasing them, while achieving a high loading capacity of up to 10 capsules per cell [139]. Moreover, researchers have discovered that plant-based, green-synthesized nanoparticles, produced through biological pathways involving living cells, offer an eco-friendly and sustainable alternative for nanoparticle fabrication. In this process, phytochemicals serve as natural stabilizers and reducing agents, facilitating the conversion of metal ions into green-synthesized metal nanoparticles. This approach can reduce biohazardous waste, improve cost-effectiveness, and enhance biocompatibility, supporting production of potent, relatively non-toxic nanoparticles [141].

Stability is another key limitation, especially for platforms designed for sustained release and prolonged half-life. NPs must maintain structural and chemical integrity in biological environments to ensure consistent efficacy and to avoid premature degradation or aggregation that compromises performance [142]. High colloidal stability, often achieved by minimizing aggregation through surface modification, is therefore essential. Among various strategies, polymeric coatings can enhance colloidal stability by creating a protective barrier that reduces agglomeration, improves dispersion in biological fluids, and preserves NPs functionality during therapy [143]. For example, hydrogel layer coating ( > 95%) has been reported to yield high colloidal stability and low protein adsorption, supporting the protein-repellent stabilizing effects of certain polymers. However, stability can be highly sensitive to environmental conditions, where exposure to salts or extreme pH may compromise stability and promote aggregation. Consequently, storage conditions play a critical role in maintaining colloidal stability and overall performance [143]. Collectively, NP evaluation should prioritize both therapeutic objectives and patient safety.

Cost is an additional barrier. Some NP materials and their intended applications are more expensive than conventional immunosuppressive treatments, limiting access even when clinical benefits may be substantial. Technical barriers during NP preparation also complicate scale-up and mass production of precisely engineered NPs, further increasing cost [136]. For example, large-scale exosome production is challenging because exosomes derived from identical cell sources can still vary in content and bioactivity due to differences in growth conditions (e.g., temperature, media composition, and supplements), which affects yield and function [122]. Improving affordability and accessibility will require standardized, safe manufacturing procedures and reproducible engineering workflows to enable reliable, scalable production of stable NP systems [46].

Some surface-modification approaches introduce additional challenges. PEGylation, which is often used to prolong circulation by reducing immune recognition, can induce anti-PEG antibodies [144]. Anti-PEG antibodies can accelerate clearance and shorten circulation time, thereby diminishing therapeutic benefit [144]. More broadly, limited modification strategies and intrinsic nanomaterial properties may contribute to resistance over time—for example, antimicrobial nanomaterials could potentially promote microbial adaptation [145]. For patients requiring long-term therapy, the risk of developing antibodies or resistance highlights the need for a broader repertoire of surface modifications that preserve efficacy while reducing these risks.

Beyond patient safety, environmental impact must also be considered. NP release into the environment could lead to accumulation in the food chain and ecological toxicity, requiring careful evaluation [146]. This motivates the development of specific disposal regulations and continuous regulatory monitoring across manufacturers and consumers. Such measures could enable real-time data collection on nanomedical waste and promote responsible management to support sustainable use of nanotechnology [147].

Despite generally favorable biocompatibility in many contexts, metallic NPs raise specific toxicity concerns. While increased surface area and dense ligand presentation can enhance local drug concentration and enable sustained, controlled release, these same features can intensify chemical interactions with host cells and increase toxicity [148]. Moreover, metallic NPs (particularly silver and gold) are often associated with antimicrobial activity, which may raise concerns about promoting microbial resistance or biofilm-associated tolerance [149]. One proposed mitigation strategy is conjugation of nitric oxide (NO) to metallic NPs [145]. In one study, NO release limited bacterial virulence, and bacterial resistance to NO has not been reported to date [145]. However, this approach may not be broadly applicable across all bacteria, and additional strategies are needed to reduce resistance risk.

Finally, long-term consequences of NP exposure can limit clinical adoption. Non-biodegradable NPs, while beneficial for sustained delivery, may accumulate in tissues and induce dysfunction or inflammation, potentially exacerbating autoimmune pathology [136]. Even when NPs are intended to traverse the blood–brain barrier (BBB) for CNS delivery, neurotoxicity remains a concern: NPs can activate microglia, promote neuroinflammation, trigger excitotoxicity, and accumulate in neural tissue even without brain-targeting ligands. These effects can lead to oxidative stress, DNA injury, and apoptosis [79]. Therefore, CNS-oriented NPs should ideally be designed for degradability in neural environments and engineered to minimize microglial activation [79]. In addition, NP exposure may alter BBB integrity and inadvertently increase uptake of toxic substances by increasing BBB permeability [79].

Inter-individual variability also complicates safety. For example, acute hypersensitivity reactions to USPIO have been reported, requiring careful clinical use—particularly in patients with immune-related diseases, where autoreactive cells may contribute to hypersensitivity [47]. Reassuringly, such adverse events appear rare: among 1.2 million injections, 79 cases of anaphylaxis were reported, with most neither severe nor fatal, and moderate side effects observed in only 0.2% of cases [47]. Nonetheless, further optimization and risk-reduction strategies remain important.

Overall, while many NP properties can provide short-term benefits, uncertainties remain due to limited long-term evidence. Accordingly, NP design and experimental studies should explicitly balance benefits against risks to maximize clinical effectiveness while prioritizing patient safety.

A non-exhaustive list of the different NPs is provided in Table 1.

**Table 1:** Comparison of the NPs.

Types of nanoparticles	Advantages	Disadvantages	Safety concerns/Limitations	Possible disease application
Iron-oxide nanoparticles (IONPs)	1. Strengthen signals in imaging [44]. 2. High sensitivity in capturing mild pathological changes for early detection [38]. 3. Biodegradable which lowers potential health complications related to using imaging contrasting agents [38]. 4. High permeability to pass through highly selective barriers such as blood brain barrier (BBB) [51].	1. Limited staining methodology [51]. 2. Potential environmental pollution as metal-nanoparticles [146]. 3. Potential acute hypersensitivity allergic reactions [47].	1. Size [104]. 2. Concentration [139]. 3. Stability and potential for aggregation [142]. 4. Storage environment [143]. 5. High cost for large-scale production [136]. 6. Standardized safe manufacturing procedures to minimize therapeutic discrepancies between batches [122]. 7. Potential antibodies arising against limited modifications to nanoparticles [145]. 8. Non-biodegradability that causes potential accumulation at undesired target area [79].	T1D [38], MS [51]
Gold nanoparticles (GNPs)	1. Inert nature, contributing to high stability, lowering risk of undesired cell-nanoparticle interaction [15]. 2. Anti-inflammatory and antioxidant [15]. 3. Anti-microbial [15]. 4. Highly sensitive, contributed by surface plasmon resonance (SPR), offering a more convenient self-monitoring option [53]. 5. Low cytotoxicity [15]. 6. High permeability to pass through highly selective barriers such as BBB [79]. 7. Highly sensitive for early detection [55].	1. Potential microbial resistance [149]. 2. Potential environmental pollution as metal-nanoparticles [146]. 3. More costly^156^.		RA [53], celiac disease [15], MS [15]
Silver nanoparticles (AgNPs)	1. Anti-microbial [56]. 2. Strengthen signals and improve sensitivity of current diagnostic technology [58]. 3. Antioxidant [84–86].	1. Possibility to stimulate ROS generation [84–86]. 2. Potential microbial resistance [149]. 3.Potential environmental pollution as metal-nanoparticles [146].		MS [58], RA [84]
Liposomes	1.High specificity in desired target site [60]. 2. Minimizing unwanted accumulation off-target site [60, 118]. 3. Strengthen signals [61]. 4. High biocompatible with its hydrophobic core and hydrophilic outer layer to deliver both soluble and insoluble components to target site [93] 5. Cationic nature to pass through highly selective barriers such as BBB [79]. 6. Increasing drug stability [80]. 7. Antibacterial [98].	1. Cationic nanoparticles exhibit more toxicity than anionic nanoparticles [79]. 2. Retention of liposomes [103].		IBD [60], RA [61, 103], T1D [113], MS [103, 113]
Exosomes	1. Encapsulated content aids in early detection of disease [62]. 2. High reflective of therapeutic outcome and disease progression [62]. 3. Highly biocompatible and permeable to pass through highly selective barriers such as BBB [80]. 4. Low immunogenicity [123]. 5. Unique compositions since they are produced from different parental cells [120, 121]. 6. Anti-inflammatory and antioxidant [119].	1. Low yield, purification challenges and difficulty in storage [62, 122]. 2. Exogenous modification challenges membrane integrity, potentially lowering its therapeutic effect [130]. 3. Difficulty in monitoring interaction between exosomes and target cells and tissues [131].		RA [64, 128], SS [62], MS [119, 120, 126, 127, 129], T1D
Chitosan-based nanoparticles	1. Low nanotoxicity, high biocompatibility and biodegradability [106]. 2. Enhancing half-life and bioavailability of drugs [97]. 3. Cationic nature increases drug absorption [98, 99]. 4. Antioxidant and anti-bacterial.	1. Cationic nanoparticles exhibit more toxicity than anionic nanoparticles [79].		MS [102], RA [96]
PLGA/PLA-Based nanoparticles (PLGA NPs)	1. High stability in encapsulating drugs, allowing sustained drug release at desired therapeutic threshold concentration [98]. 2. High biocompatibility and biodegradable with low nanotoxicity [104]. 3. High specificity and strong encapsulating abilities, preventing unwanted interaction with off target cells [76].	1. Potential nanotoxicity and adverse health complications [70].		UC [80], SLE [110–112], MS [103]
Carbon-based nanoparticles	1. Good electrical conductivity, intrinsic fluorescence and high photostability to strengthen signals [66]. 2. Cost-effective in consistent monitoring [69]. 3. Lowered nanotoxicity by absorbing compounds such as drugs, toxins and metabolites within bloodstream [135]. 4. Anti-inflammatory [67].	1. Potential nanotoxicity and adverse health complications [70].		RA [68], T1D [67]

## Conclusion and perspectives

Currently, liposomes, polymeric NPs and IONPs are among the most commonly explored platforms for the diagnosis and treatment of autoimmune disease [46]. When developing nanomaterials for in vivo use in patients with autoimmune disease, three immune-related consequences should be considered [15]. First, it is critical to avoid immune-mediated clearance, in which defensive immune responses eliminate administered NPs, thereby reducing diagnostic and therapeutic efficacy [15]. Second, certain nanomaterials may cause immunotoxicity, impairing immune function rather than providing benefit [15]. Third, NPs should exhibit high biocompatibility, also known as immune compatibility, to minimize disruption of normal immune responses [15].

Furthermore, early manifestations of autoimmune disease are often overlooked because symptoms can either be asymptomatic or insignificant at the initial stage. NPs such as IONPs can also be incorporated into point-of-care devices to improve biomarker sensitivity while reducing false detection, allowing rapid and convenient self-testing. For instance, anti-CCP, a relatively specific biomarker, can be detected up to 10 years before the onset of RA symptoms, offering a valuable therapeutic window [150]. However, many diagnostic tests remain difficult to access without hospital-based facilities. This limitation may be mitigated by microdevice immunoassay conjugated with MNPs [150]. In this approach, anti-CCP in plasma binds MNPs and aggregates to produce detectable signal, with performance reported to be substantially faster and more sensitive than conventional ELISA [150]. Binding specificity can also be verified by transmission electron microscopy (TEM), confirming association of MNPs with the intended biomarker [150]. Such self-diagnostic tools could reduce healthcare system burden and facilitate earlier detection with greater convenience.

Beyond diagnosis, autoimmune diseases are frequently accompanied by chronic health complications, highlighting the need for early and consistent monitoring. These complications may reflect deteriorating baseline health, immune dysregulation, suboptimal therapeutic responses, or the cumulative burden of repeated invasive procedures. Thus, long-term disease management requires diagnostic and monitoring approaches that are safe and acceptable for repeated use. In this context, NP-based platforms spanning diagnosis, monitoring, and treatment illustrate the broader promise of theranostics, where a single material system can support multiple clinical goals.

Importantly, NPs may serve not only as drug carriers but also as immunomodulatory therapies. Drug-free NPs composed of selected anti-inflammatory nanomaterials, especially when functionalized with appropriate ligands—could reduce reliance on cytotoxic drugs and potentially mitigate long-term issues such as tolerance, escalating doses, and cumulative side effects [151]. Biodegradable, drug-free NPs may help promote immune self-tolerance by targeting innate immune cells (e.g., inflammatory monocytes and neutrophils) to restrain excessive inflammation, thereby supporting a more durable and self-sustaining approach to disease control.

Immunomodulatory NPs are particularly relevant given the rising prevalence and chronic burden of diseases such as RA, which is associated with persistent inflammation and pain in small joints. However, despite encouraging preclinical results, clinical translation remains limited and will likely require combined strategies that address both upstream immune dysregulation (to induce tolerance) and ongoing symptoms. For instance, an in vivo study in RA models used poly(lactic-co-glycolic acid)–poly(ethylene glycol)-maleimide (PLGA-PEG-MAL) NPs conjugated with joint-relevant peptide antigens (aggrecan_70-84_ and type II bovine collagen_256-270_) and encapsulating calcitriol [152]. This approach reportedly promoted tolerogenic dendritic cells, while calcitriol reduced disease severity, reflected by improved clinical scores, reduced cartilage proteoglycan loss, and decreased bone damage, supported by high-resolution microcomputed tomography and histomorphometry analysis [152]. Although such strategies are promising, the evidence base remains limited, and additional studies, including clinical trials, are needed to establish feasibility, safety, and durability of benefit in humans.

Overall, while NP-based approaches show strong potential for the diagnosis and treatment of autoimmune disease, further work is required to confirm clinical safety and effectiveness, as many studies remain at the animal-model stage. Expanded clinical trial evidence would increase patient and clinician confidence in NP applications. Meanwhile, key challenges, such as scalable standardization, stability, and potential cytotoxicity, continue to be actively addressed. As these hurdles are overcome, nanotechnology may substantially advance how autoimmune diseases are detected, monitored, and treated.

## Supplementary information

Supp Table 1
